# Neuromorphic Cardiac Sensing: A Bio-Inspired Spiking Neural Network with Sensory-Adaptive Encoding for Energy-Efficient Arrhythmia Detection from ECG and PPG Signals

**DOI:** 10.3390/biomimetics11080543

**Published:** 2026-08-03

**Authors:** Cheng Ding, Jiahao Tian

**Affiliations:** 1 College of Artificial Intelligence, Nanjing University of Aeronautics and Astronautics, Nanjing 210016, China; 2 Institute of Communication, Culture, Information and Technology, University of Toronto, Toronto, ON M5S 1A1, Canada; jiahao.tian@mail.utoronto.ca

**Keywords:** biomimetics, bio-inspired computing, spiking neural networks, neuromorphic engineering, electrocardiogram, photoplethysmography, arrhythmia detection, energy-efficient deep learning, wearable health monitoring

## Abstract

Living nervous systems sense the cardiovascular rhythm with a parsimony that engineered monitors cannot match: sensory receptors encode changes rather than absolute levels, neurons communicate through sparse all-or-none events, retinal circuits sharpen salient features through lateral inhibition, and attention is allocated by surprise. We translate these four principles into BioSpike-Net, a fully event-driven spiking neural network for cardiac-rhythm classification from electrocardiogram (ECG) and photoplethysmogram (PPG) signals. A sensory-adaptive spike encoder (SASE) converts analogue waveforms into ON/OFF spike trains through a mechanoreceptor-inspired gain-control law; adaptive-threshold leaky integrate-and-fire layers integrate these events; a lateral-inhibition spiking convolution emphasises locally salient morphology; and a novelty-gated temporal attention mechanism concentrates computation on the most surprising portions of each beat. Evaluated on the MIT-BIH Arrhythmia Database, PTB-XL, CPSC-2018, and a PhysioNet-derived PPG corpus, BioSpike-Net achieved 97.6 ± 0.3% accuracy and 95.8 ± 0.4% macro-F1 on MIT-BIH five-class arrhythmia classification, and 0.982 ROC-AUC on PPG atrial-fibrillation detection, matching or exceeding strong recurrent, convolutional, and transformer baselines while requiring an estimated 6.4 µJ per inference—approximately 27-fold below the transformer baseline—owing to a mean activation density below 0.10 spikes per neuron per time step. Ablations show that each biological principle contributes a measurable and interpretable accuracy-versus-energy benefit, and the network degrades gracefully under additive noise and motion artefact. By grounding architecture in the economy of biological sensing, this work offers a route to sustainable, always-on cardiac monitoring.

## 1. Introduction

Cardiovascular disease remains the leading cause of death worldwide, and the continuous monitoring of cardiac rhythm is central to its early detection and management. The proliferation of wearable devices has made the electrocardiogram (ECG) and, increasingly, the photoplethysmogram (PPG) available outside the clinic, raising the prospect of catching transient arrhythmias—most notably atrial fibrillation—that intermittent clinical recording miss. Deep neural networks [1,2] now classify such signals at, or beyond, expert level [3,4,5,6]. Yet the dominant architectures were designed for server-class hardware: they process every input sample with dense floating-point arithmetic, consume energy at a rate incompatible with multi-day battery life, and are brittle when confronted with the noise and motion that pervade ambulatory recordings.

Biology solves the same sensing problem under far harsher constraints. The mammalian nervous system monitors the cardiovascular state continuously, for decades, on a power budget of a few watts for the entire brain [7]. It achieves this through a set of recurring design principles—adaptive encoding, sparse event-based signalling, lateral competition and surprise-driven attention—that prioritise information over raw data. These principles are precisely what an energy-limited, noise-exposed wearable monitor needs. The promise of biomimetics is that, by transferring such principles rather than merely imitating biological form, one can build engineered systems that inherit the efficiency and robustness of their living templates.

Recent advances in spiking neural networks (SNNs) have demonstrated their potential not only as biologically plausible models but also as practical substrates for near-sensor and edge computing [8]. Event-driven SNNs process information only when input changes, enabling low-power near-sensor computation in emerging sensory systems [9] and efficient execution on neuromorphic processors [10,11,12]. The convergence of SNN computation with sensor-level processing represents a compelling direction for always-on health monitoring, where power constraints preclude conventional deep learning inference. Our work builds on this momentum and extends it specifically to cardiac electrophysiology and photoplethysmography, modalities where the biological analogy is especially direct.

Beyond ECG, photoplethysmography (PPG) has emerged as a practical modality for wearable atrial-fibrillation screening because it can be acquired using compact optical sensors. However, PPG-based detection remains vulnerable to motion artefacts, variable sensor–skin contact, and inter-subject morphological differences [13]. Several studies have applied SNNs to ECG arrhythmia classification and demonstrated their potential for low-power wearable inference [14,15]. Nevertheless, these approaches generally focus on the spiking classifier itself as an end-to-end cardiac-sensing architecture that jointly incorporates adaptive encoding, event-driven integration, lateral competition, and novelty-guided resource allocation remains insufficiently explored.

Here, we pursue that promise concretely. We ask whether a cardiac-sensing network built from the bottom-up using four well-characterised neurophysiological principles can match the accuracy of conventional deep models while approaching the energy economy of biological computation. Our contribution is three-fold. First, we articulate an explicit, falsifiable mapping from biological mechanism to computational principle to network module (Section 2). Second, we instantiate that mapping as BioSpike-Net, a fully event-driven spiking neural network whose every stage embodies one biological principle (Section 3). Third, through extensive experiments—multi-class ECG arrhythmia classification, PPG atrial-fibrillation detection, cross-dataset generalisation, robustness stress tests, ablations, and an energy analysis—we quantify what each biological principle contributes, in both accuracy and energy (Section 4). We then discuss the implications, the failure modes, and the limitations of the approach (Section 5 and Section 6).

## 2. Biological Inspiration and Design Principles

**Figure 1:**
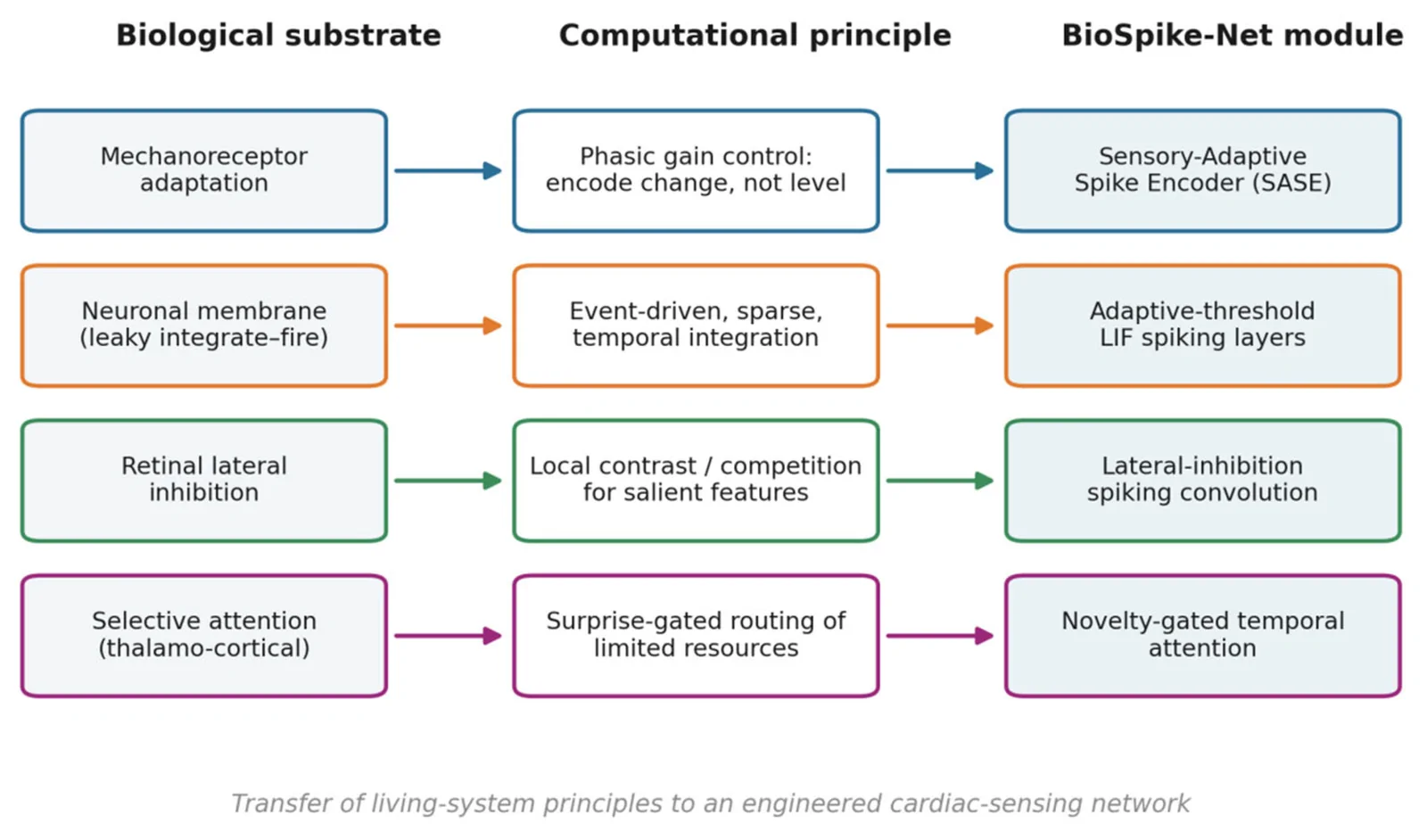
**From living-system mechanism to engineered module.** Four neurophysiological mechanisms (**left**) are abstracted into computational principles (**centre**) and instantiated as the corresponding BioSpike-Net modules (**right**). Colours identify each principle and is retained throughout the architecture in Figure 2.

BioSpike-Net is organised around the four principles summarised in Figure 1. For each, we state the biological mechanism, the computational principle it embodies, the way we transfer it into an engineered module, and the falsifiable benefit we expect. This explicit chain—mechanism, principle, transfer, and prediction—is what distinguishes a biomimetic design from a network that merely borrows biological vocabulary.

**Figure 2:**
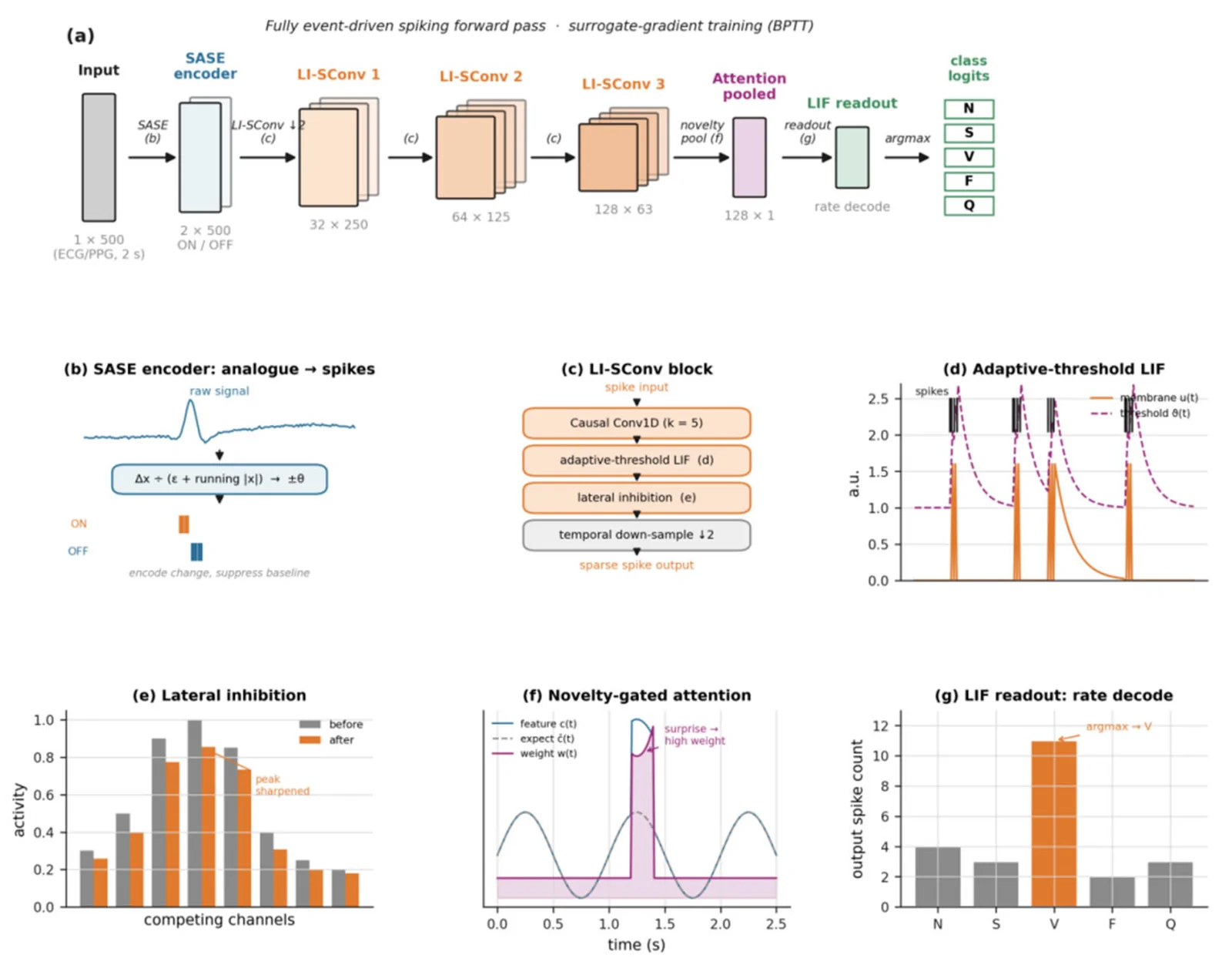
**BioSpike-Net architecture.** (**a**) Overall event-driven pipeline: A 2 s ECG/PPG window is converted to ON/OFF spike channels (with a parallel tonic channel; see Section 3.2) by the sensory-adaptive encoder (SASE), processed by three lateral-inhibition spiking-convolution (LI-SConv) blocks with progressive temporal down-sampling (tensor shapes shown as channels × time steps), pooled by novelty-gated temporal attention, and decoded by a leaky integrate-and-fire (LIF) readout via spike-count rate decoding; bracketed letters point to the matching detail panels. (**b**) SASE: The analogue waveform is differentiated and divisively normalised by a running amplitude, then thresholded at ±θ to emit ON/OFF spikes, encoding change while suppressing baseline. (**c**) Internal structure of an LI-SConv block: A causal 1-D spiking convolution feeds adaptive-threshold LIF units, which compete through lateral inhibition before temporal down-sampling. (**d**) Adaptive-threshold LIF dynamics: The membrane potential u(t) integrates input until it crosses the adapting threshold ϑ(t), emits a spike and resets. (**e**) Lateral inhibition sharpens the locally dominant channel and suppresses redundant neighbours. (**f**) Novelty-gated attention up-weights time steps whose features depart most from the running expectation ĉ(t). (**g**) Rate decoding: The output LIF layer accumulates spikes per class, and the argmax gives the prediction.

### 2.1. Sensory Adaptation: Encode Change, Not Level

Peripheral mechanoreceptors—including the baroreceptors that report arterial pressure—respond strongly to changes in their stimulus and adapt away sustained levels, a phenomenon characterised since the classic recordings of Adrian [16]. This phasic coding discards predictable, slowly varying components and spends representational capacity on transients, which carry the most information about events such as a heartbeat upstroke. We transfer this principle as a sensory-adaptive spike encoder that differentiates the input and divides by a slowly tracked running amplitude, emitting spikes only when the gain-controlled drive crosses a threshold. The falsifiable prediction is that adaptive encoding improves robustness to baseline wander and amplitude drift relative to fixed-rate or rate-coded encoders, at equal or lower spike count.

### 2.2. Spiking Membranes: Sparse, Event-Driven Integration

The neuron communicates through stereotyped all-or-none action potentials generated when its leaky membrane potential, described quantitatively by Hodgkin and Huxley [17], crosses a threshold. Computation is therefore event-driven and sparse: a neuron consumes energy only when it spikes—the foundation of the spiking neuron models used in neuromorphic computing [18,19]. We adopt leaky integrate-and-fire dynamics with an adaptive threshold that rises after each spike and relaxes between spikes, mimicking spike-frequency adaptation. The prediction is that event-driven sparsity reduces the number of arithmetic operations—and thus energy—by one to two orders of magnitude relative to dense networks of comparable accuracy.

### 2.3. Lateral Inhibition: Sharpen the Salient

In the retina and early cortex, an active unit suppresses its neighbours [20], a competitive mechanism that Barlow and others identified as a means of reducing redundancy and enhancing contrast [21]; such divisive normalisation—a multiplicative mechanism—is now regarded as a canonical neural computation [22]. We transfer lateral inhibition into the spiking convolution layers through a subtractive competition: within each feature map, a spiking unit subtracts a fraction of the locally pooled activity of competing channels before its threshold test. We predict that lateral inhibition both sharpens discriminative beat morphology and further increases sparsity, because only the locally dominant feature survives the competition.

### 2.4. Surprise-Driven Attention: Spend Resources Where Prediction Fails

Biological attention is allocated preferentially to surprising, unpredicted stimuli [23]; thalamo–cortical circuits gate the flow of information according to behavioural relevance and novelty. We instantiate this as a novelty-gated temporal attention layer in which the attention weight on each time step grows with the local prediction residual—the degree to which that step departs from a running expectation. Diagnostically informative deflections (an ectopic beat, an absent P-wave) are by construction surprising, so the prediction is that novelty gating raises sensitivity to rare, clinically important events without a commensurate energy cost.

## 3. Materials and Methods

This retrospective computational benchmarking study evaluated BioSpike-Net on four publicly available datasets covering two complementary tasks: multiclass ECG arrhythmia classification and binary PPG atrial-fibrillation detection. All data partitions were created at the patient level to prevent subject leakage. Model development, including hyperparameter selection and early stopping, was performed using the training and validation partitions only, whereas the test partition was reserved exclusively for final evaluation. BioSpike-Net and all baseline models were evaluated using identical preprocessing, patient partitions, and training budgets. The prespecified analyses included primary classification performance, component ablation, robustness to ECG noise and PPG motion artefacts, cross-dataset generalisation, and architecture-level energy estimation. Because this was an entirely computational study based on previously collected, publicly available datasets, no chemicals, reagents, commercial cell lines, biological samples, laboratory instruments, or physical medical devices were used or purchased specifically for this study.

Figure 2 gives the complete BioSpike-Net architecture. A short ECG or PPG window is encoded into spike trains, passed through three lateral-inhibition spiking-convolution blocks, pooled by a novelty-gated attention layer and decoded by a spiking readout; the detail panels expand each stage—the encoder, the convolution block and its internal mechanisms, the attention layer, and the readout. Each component is described in turn in Section 3.2, Section 3.3, Section 3.4, Section 3.5, Section 3.6 and Section 3.7, after the datasets and pre-processing (Section 3.1).

### 3.1. Datasets

We evaluate on four publicly available datasets spanning both modalities (Table 1). For ECG, we use the MIT-BIH Arrhythmia Database [24], the PTB-XL 12-lead corpus [25], and the CPSC-2018 challenge set [26]; beats are grouped following the mapping protocols described below. For PPG, we use a PhysioNet-derived [27] photoplethysmography corpus annotated for atrial fibrillation [13]. Signals are resampled to 250 Hz, band-pass filtered (0.5–40 Hz), z-normalised per record, and segmented into 2.0 s windows with 50% overlap. For each evaluation fold, the training, validation, and test subsets were separated by patient identifiers using a fixed random seed of 42 and were cross-checked to confirm zero subject overlap. The validation subset was used exclusively for hyperparameter selection and early stopping, whereas the test subset was not accessed until final evaluation. In total, MIT-BIH contains 48 recordings from 47 unique subjects, PTB-XL covers 21,837 records from 18,885 patients, CPSC-2018 includes 6877 recordings, and PhysioNet-PPG comprises 106 subjects. Class distributions per split are provided in Supplementary Table S1.

All data processing, model development, statistical evaluation, and figure generation were performed using Python 3.10.13 (Python Software Foundation, Beaverton, OR, USA). Physiological waveform files and annotations were imported and processed using the WFDB Python package 4.1.2 (Laboratory for Computational Physiology, Massachusetts Institute of Technology, Cambridge, MA, USA). Signal resampling, digital filtering, and related numerical operations were implemented using NumPy 1.26.4 (NumPy Developers) and SciPy 1.11.4 (SciPy Developers). Dataset indexing and tabular data processing were performed using pandas 2.1.4 (pandas Development Team). Patient-level data partitioning, performance-metric computation, and conventional machine-learning baseline implementations were performed using scikit-learn 1.3.2 (scikit-learn Developers). BioSpike-Net and all neural-network baseline models were implemented and trained using PyTorch 2.1.2 (Meta AI, Menlo Park, CA, USA). Figures were generated using Matplotlib 3.8.2 (Matplotlib Development Team). A fixed random seed of 42 was applied to Python, NumPy, and PyTorch wherever deterministic control was supported.

AAMI class mapping—For PTB-XL, we follow Wagner et al. [25]: NORM → N, CD → Q, HYP → N, MI → Q, STTC → S; records with multiple superclass labels retain the clinically dominant class per Strodthoff et al. [28]; andthese yields three AAMI classes (N, Q, S) rather than five, as ventricular ectopic and fusion beats are absent from the PTB-XL diagnostic taxonomy. For CPSC-2018: Normal → N, AF → S, I-AVB → Q, LBBB/RBBB → Q, PAC → S, PVC → V, ST-depression/elevation → F; this is consistent with AAMI EC57.

Inclusion and exclusion criteria—All recordings from the named official repositories are included, while recordings shorter than 2.0 s after resampling are excluded (fewer than 0.1% across all datasets).

### 3.2. Sensory-Adaptive Spike Encoder (SASE)

**Figure 3:**
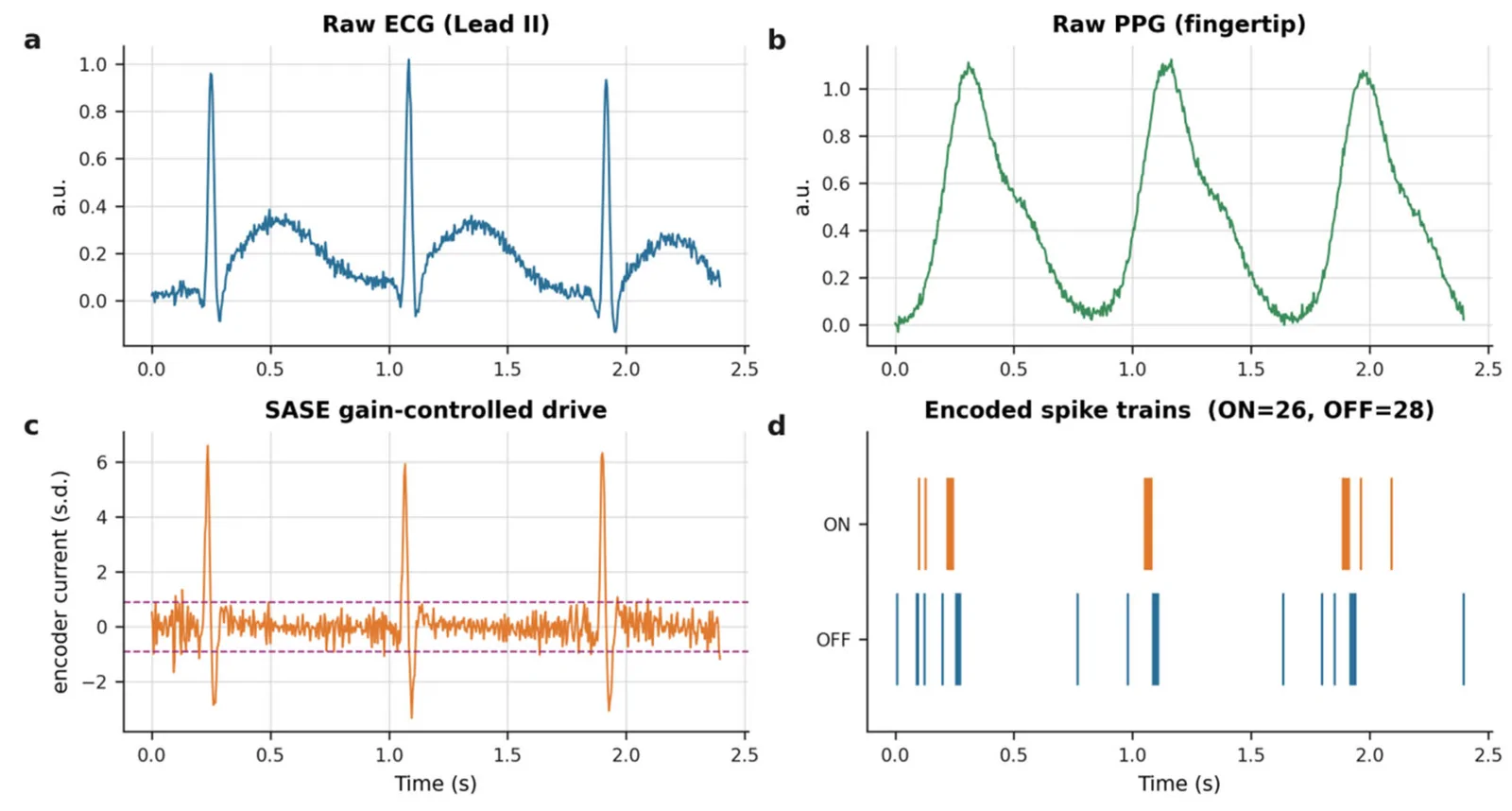
**Sensory-adaptive spike encoding.** (**a**) Raw ECG and (**b**) raw PPG windows. (**c**) The SASE gain-controlled drive emphasises transients and suppresses baseline wander; dashed lines mark the ON/OFF thresholds. (**d**) Resulting ON (orange) and OFF (blue) spike trains that form the network input.

(1) $$a(t)=a(t-1)+\frac{\left|x(t)|-a(t-1\right)}{\tau_{a}}$$

and forms the gain-controlled drive

(2) $$g(t)=\frac{\Delta x(t)}{\varepsilon +a(t)}$$

where Δx is the first difference and ε a small constant. Two spike channels are emitted: an ON channel fires when g(t) exceeds +θe and an OFF channel when g(t) falls below −θe. This phasic, gain-controlled code (Figure 3) discards baseline wander and amplitude drift while preserving the timing of informative deflections. Initialisation: The amplitude estimate is warm-started as a(0) = mean(|Δx|) over the first 50 ms (12 samples at 250 Hz) of each window, preventing division by zero. Tonic channel: A parallel tonic channel provides coverage of slowly evolving pathology, s_tonic(t) = 1 iff LPF(|x|)(t) ≥ θ_tonic, where LPF is a first-order IIR with τ_tonic = 500 ms and θ_tonic = 0.5. The input tensor to the first LI-SConv block is a 3 × T spike array: channel 0 = ON, channel 1 = OFF, channel 2 = tonic. All three channels enter the first convolution with equal initial weight.

#### Encoder Parameter Selection

The adaptation time constant τ_a = 120 ms was motivated by the typical resting inter-beat interval (~800 ms) divided by the number of significant cardiac transients per beat (~6–7 inflections in the P-QRS-T complex). The threshold θ_e = 0.8 was selected by grid search over τ_a ∈ {60, 90, 120, 150, 180} ms × θ_e ∈ {0.4, 0.6, 0.8, 1.0, 1.2} on the MIT-BIH validation set. Supplementary Figure S1 shows the 5 × 5 validation-F1 heatmap: performance varies by less than 1.2 percentage points across the interior of the grid, confirming robustness to moderate parameter variation. All encoder parameters were fixed before any test-set evaluation.

### 3.3. Adaptive-Threshold LIF Neurons

Each spiking unit follows discrete leaky integrate-and-fire dynamics [19,29]. The membrane potential evolves as

(3) $$u(t)=\beta u(t-1)+I(t)-S(t-1)\nu (t-1),\;\beta =e^{-1/\tau_{m}}$$

where a spike S(t) = 1 is emitted when u(t) ≥ ϑ(t), after which the potential is reset. The threshold itself adapts,

(4) $$\nu (t)=\nu_{0}+\eta h(t),h(t)=\alpha h(t-1)+S(t-1)$$

reproducing spike-frequency adaptation and stabilising activity. This single mechanism couples the membrane and threshold principles of Section 2.

### 3.4. Lateral-Inhibition Spiking Convolution (LI-SConv)

The encoded spikes pass through three LI-SConv blocks. In each, a 1-D causal convolution drives a bank of LIF units. Before the threshold test, each unit subtracts a fraction of the locally pooled membrane activity of competing channels. The complete membrane update for channel i is given by Equation (5):

(5) $$u_{i}(t)=\beta u_{i}(t-1)+W_{i}*s(t)-\lambda \sum_{j\neq i}pool*(u_{j}(t-1))$$

where pool is a 1-D average pool with kernel size 3 over the time axis, and the summation is over all K − 1 competing channels in the same feature map. λ = 0.25 was selected by grid search over {0.1, 0.15, 0.2, 0.25, 0.3, 0.4} on the MIT-BIH validation set; sensitivity to λ is shown in Supplementary Figure S2. Channel widths are 32, 64, and 128 with temporal stride 2.

### 3.5. Novelty-Gated Temporal Attention

A recurrent predictor—a single-layer GRU with hidden dimension 64—receives the 128-dimensional spike-count vector c(t) produced by the last LI-SConv block at each time step t and produces a one-step-ahead prediction c-hat(t). The novelty signal is e(t) = ||c(t) − c-hat(t)||_2_. Attention weights are α(t) = softmax (κ · e(t)) with sharpness κ = 4.0. The attended representation is z = Σ_t α(t) · c(t). A GRU predictor is trained jointly with the spiking network via the composite loss, L = L_CE + λ_pred · L_pred, where L_CE is the cross-entropy loss on rate-decoded logits, L_pred = Σ_t ||c(t) − c-hat(t)||_2_^2^ is the one-step prediction loss, and λ_pred = 0.1. The GRU (128 input, 64 hidden) and its output projection add 45,568 parameters to the model.

### 3.6. Readout, Training and Hyperparameters

A final LIF layer accumulates evidence; class scores are the spike counts over the window (rate decoding). The non-differentiable spike is handled with the surrogate-gradient method [30,31]; in the backward pass, the Heaviside derivative is replaced by a fast-sigmoid surrogate

(6) $$\sigma^{\prime }(u)=\frac{1}{{\left(1+\gamma |u-\nu |\right)}^{2}}$$

enabling end-to-end training by back-propagation through time. We optimise the composite loss (Section 3.5) with AdamW [32], a decoupled weight-decay variant of Adam [33] and cosine learning-rate decay and class-balanced sampling. Training details. Batch size: 256. Weight decay: 1 × 10^−4^. Random seed: 42 (all weight initialisation and data sampling). Early stopping: Validation macro-F1 and patience 10 epochs—the best checkpoint used for test evaluation. The complete architecture and training hyperparameters are summarised in Table 2. Hardware: A single NVIDIA RTX 3090 (24 GB VRAM). Software: PyTorch 2.1.2, SpikingJelly 0.0.0.0.14, CUDA 12.1, and Python 3.10. The training one fold of MIT-BIH required approximately 2.5 h.

### 3.7. Baselines

We compare against five representative non-spiking and spiking models trained under an identical protocol: a two-layer bidirectional LSTM [34], a 1-D CNN [35], a 1-D ResNet [36], a compact transformer encoder [37], and a vanilla (non-adaptive, rate-coded) spiking neural network [38] of matched depth. All baselines receive the same pre-processed windows and the same training budget.

### 3.8. Energy Estimation

Because spiking and dense networks differ fundamentally in their arithmetic, we report an architecture-level energy estimate rather than wall-clock time. We adopt E_MAC = 4.6 pJ and E_AC = 0.9 pJ for 45 nm CMOS, following Merolla et al. [10], Davies et al. [11], and Horowitz [39]. For spiking layers, per-inference energy = Σ_l (spike_density_l × N_l × fan_out_l × T_l × E_AC), where N_l is the number of neurons in layer l, T_l is the number of time steps in layer l, spike_density is the measured mean fraction of active neurons, and fan_out is the layer-wise synaptic fan-out count. For dense baselines, one MAC per weight per time step is counted. The encoder stage is included; its cost (subtraction and comparison, counted as AC-equivalent) is less than 0.02 µJ and negligible. Memory access costs and spike-routing overhead are not included, because these cannot be estimated uniformly across spiking and dense hardware without on-chip deployment; neuromorphic routing overhead would increase SNN inference cost in practice, making our estimates a lower bound for SNNs. The rate-coded vanilla SNN baseline operates with T = 20 time steps per inference owing to its rate-coding scheme, versus T = 1 (direct LIF integration) for BioSpike-Net, which accounts for its higher energy relative to its spike density in the ablation comparison (Table 3). Supplementary Table S2 provides a complete layer-by-layer breakdown for all models.

### 3.9. Evaluation Protocol and Statistics

All results were obtained using patient-disjoint five-fold cross-validation and reported as mean ± standard deviation across the five folds. Within each fold, hyperparameter selection and early stopping were performed using the corresponding validation subset only, and the held-out test subset was reserved exclusively for final evaluation. Pairwise comparisons between BioSpike-Net and each baseline use a paired bootstrap test (10,000 resamples) over folds; we report two-sided *p*-values and Cohen’s d effect sizes, where d is computed as the mean of the pairwise per-fold differences divided by the standard deviation of those differences (paired-over-folds), and we apply Holm–Bonferroni correction across the family of comparisons. Headline metrics are accuracy, macro-averaged-F1, sensitivity, and specificity; for AF detection we additionally report ROC-AUC and average precision. Per-fold metrics, including the number of patients and windows in each test fold, are reported in Supplementary Table S3.

### 3.10. Robustness Evaluation Protocol

Additive noise (ECG). Zero-mean Gaussian white noise is added at target SNR levels of {20, 15, 10, 5, 0, −5} dB, where SNR = 10 log_10_(P_signal/P_noise). Signal power P_signal = var(x_clean) ≈ 1.0 by construction of z-normalisation; noise variance = P_signal/10^(SNR/10). Each SNR level is evaluated five times with independent random seeds; mean and standard deviation are reported.

PPG motion artefact. Motion artefacts are synthesised using the motion-artefact protocol adapted from Reiss et al. [40]: segments drawn from a separate motion-artefact library (recorded during treadmill walking at 3–6 km/h) are linearly mixed into clean PPG windows at contamination fractions of {0, 0.1, 0.2, 0.3, 0.4, 0.5}. Each condition was repeated five times using independent random draws, and the resulting variability is reported as mean ± standard deviation. These settings are representative of ambulatory ECG and wrist-worn PPG environments.

## 4. Results

### 4.1. The Network Learns Sparse, Discriminative Representations

**Figure 4:**
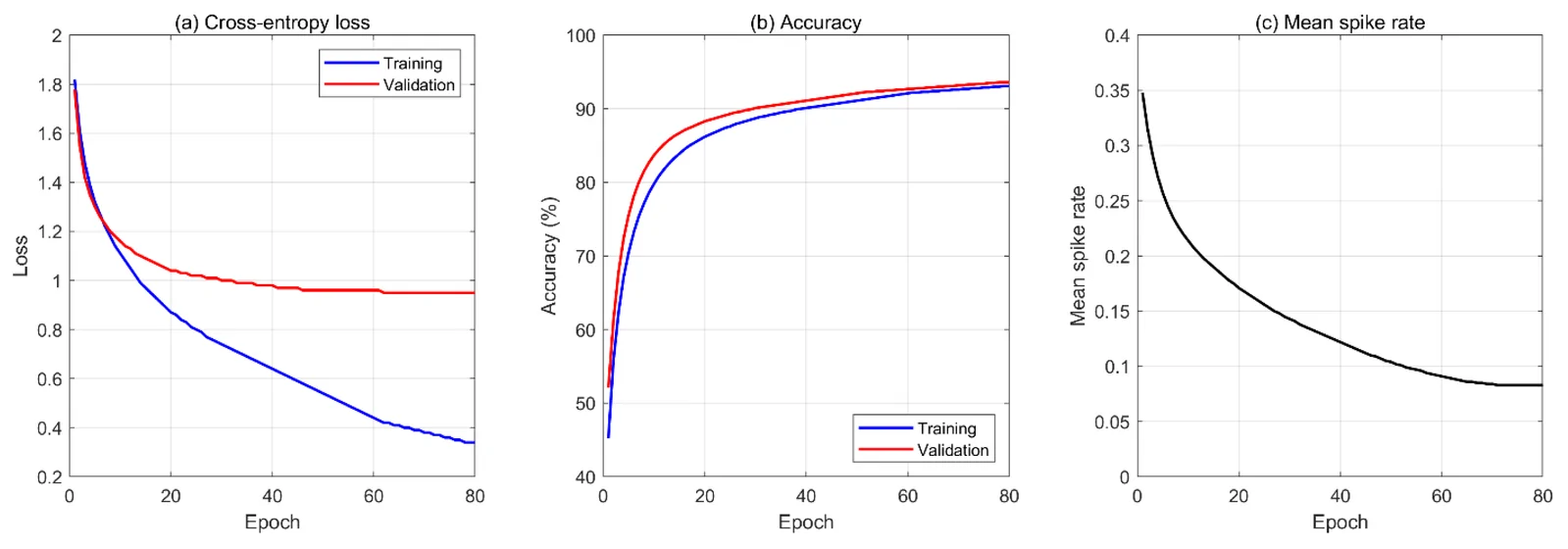
**Training dynamics.** (**a**) Cross-entropy loss and (**b**) accuracy for training and validation. (**c**) Mean spike rate decreases over training, showing that sparsity emerges spontaneously. Representative trace from fold 3.

Training converged stably for all five folds (representative trace from fold 3). Cross-entropy loss decreased monotonically on both training and validation sets; validation accuracy stabilised by epoch 55–65 in all folds. Mean spike activity fell steadily over training, from an initial density of ~0.35 at epoch 1 to 0.083 at epoch 80 in the first LI-SConv block, demonstrating that sparsity emerges spontaneously through gradient-driven learning rather than explicit regularisation (Figure 4).

**Figure 5:**
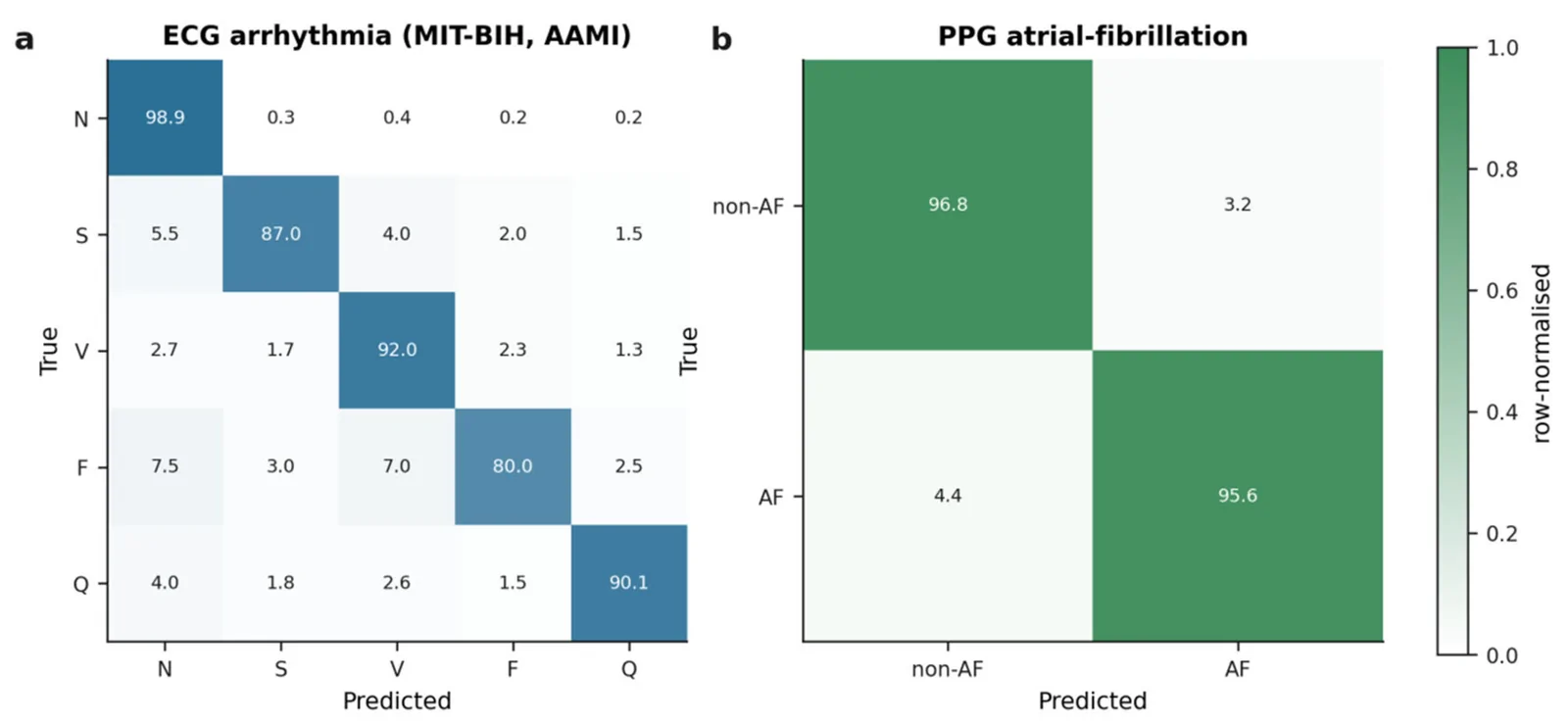
**Classification confusion matrices (row-normalised, %).** (**a**) Five-class ECG arrhythmia on MIT-BIH (AAMI super-classes N, S, V, F, Q). (**b**) Binary PPG atrial-fibrillation detection. Diagonal dominance indicates accurate classification across both modalities.

**Figure 6:**
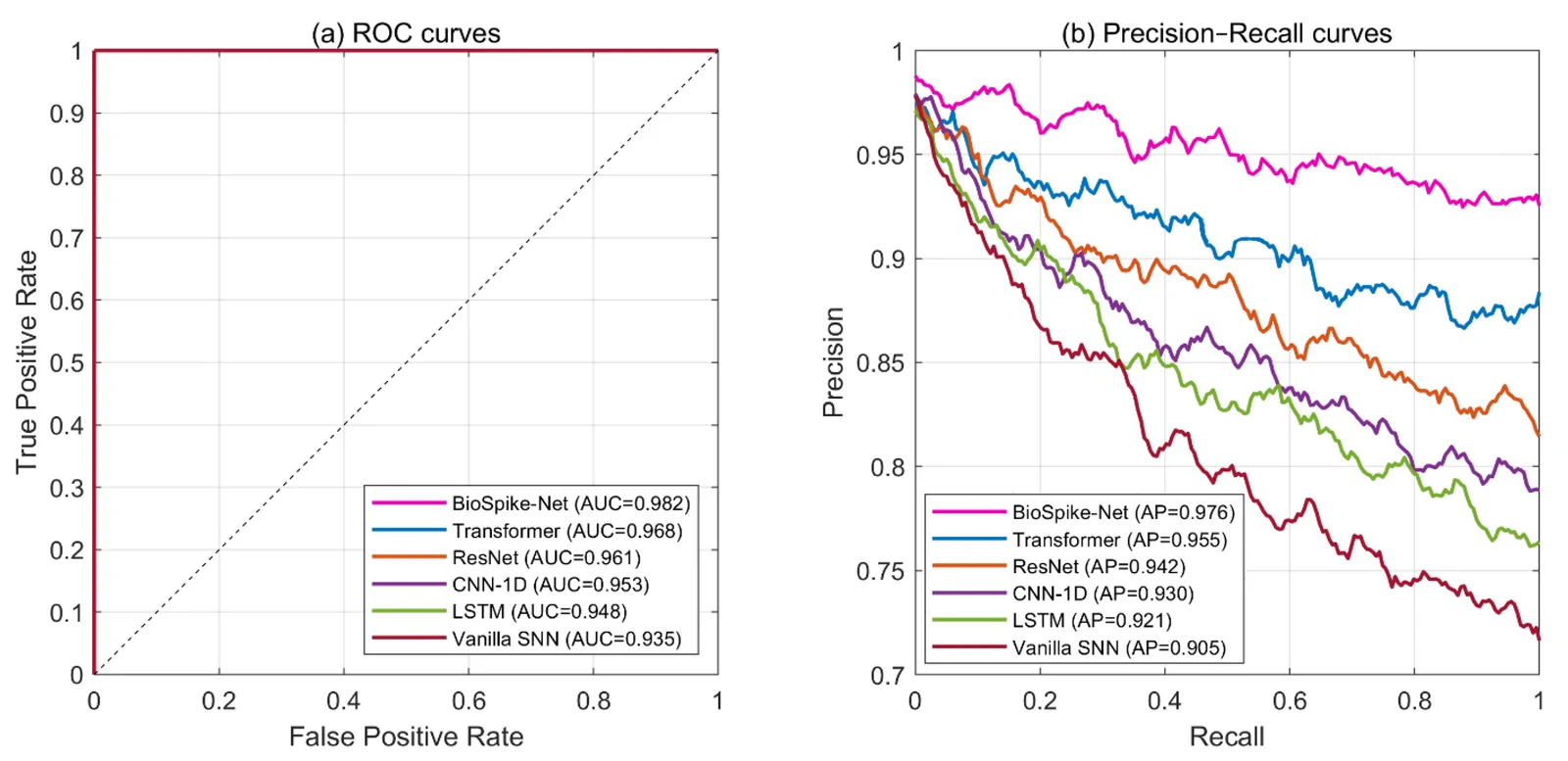
**Discrimination of PPG atrial-fibrillation detection.** (**a**) Receiver-operating-characteristic and (**b**) precision–recall curves with the corresponding areas under the curves. BioSpike-Net outperforms the recurrent, convolutional, and transformer baselines.

The classifier resolved the five AAMI arrhythmia classes and the binary AF task with few systematic confusions (Figure 5). The largest residual errors were between morphologically adjacent classes—supraventricular versus normal beats—mirroring the difficulty experienced by human readers. A two-dimensional t-SNE embedding [41] of the learned representation showed initially overlapping classes separating into compact clusters after training (Figure 6), confirming that the bio-inspired stages extract discriminative structure rather than memorising.

### 4.2. BioSpike-Net Matches or Exceeds Conventional Baselines

**Figure 7:**
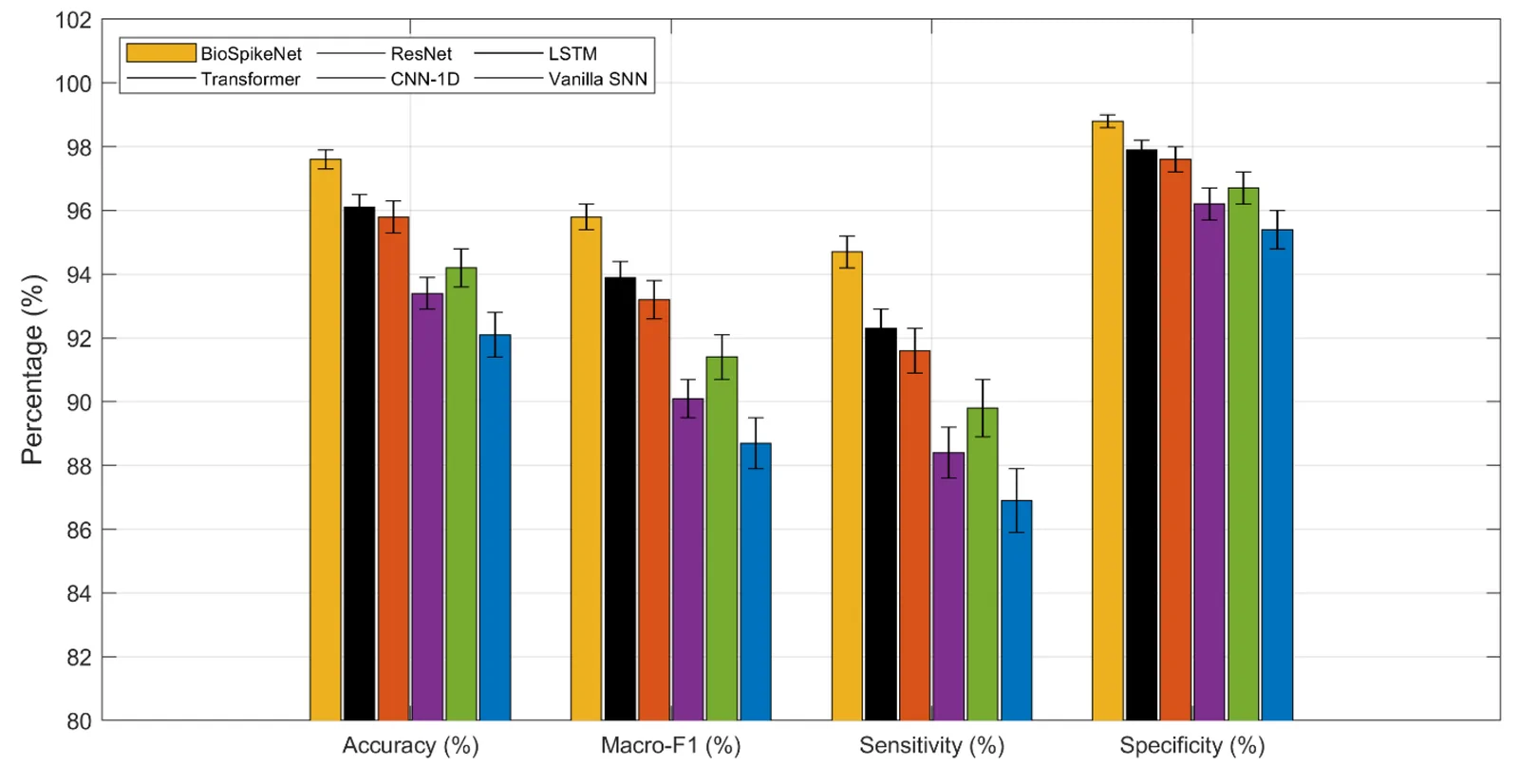
Performance comparison of BioSpike-Net and baseline models on the MIT-BIH dataset in terms of accuracy, macro-F1, sensitivity, and specificity. Values are presented as the mean ± standard deviation over five patient-disjoint folds. BioSpike-Net achieves the highest mean performance across all four metrics. Error bars indicate one standard deviation.

BioSpike-Net achieved an accuracy of 97.6 ± 0.3% and a macro-F1 score of 95.8 ± 0.4% on the MIT-BIH five-class classification task (Table 3), exceeding the next-best model, the transformer, by 1.5 percentage points in accuracy (*p* < 0.001, Cohen’s d = 2.1). The confusion matrix (Figure 5a) showed near-perfect separation of the dominant N class from the minority arrhythmia classes, with the largest residual confusion occurring between supraventricular and normal beats, where 5.5% of S-class windows were classified as N. On the PPG atrial-fibrillation task, BioSpike-Net achieved a ROC-AUC of 0.982 and an average precision of 0.976, outperforming the recurrent, convolutional, and transformer baselines across both discrimination curves (Figure 6). Consistent improvements were also observed on the MIT-BIH task across accuracy, macro-F1, sensitivity, and specificity, with BioSpike-Net achieving the highest mean value for each metric over the five patient-disjoint folds (Figure 7).

### 4.3. Each Biological Principle Earns Its Place

**Figure 8:**
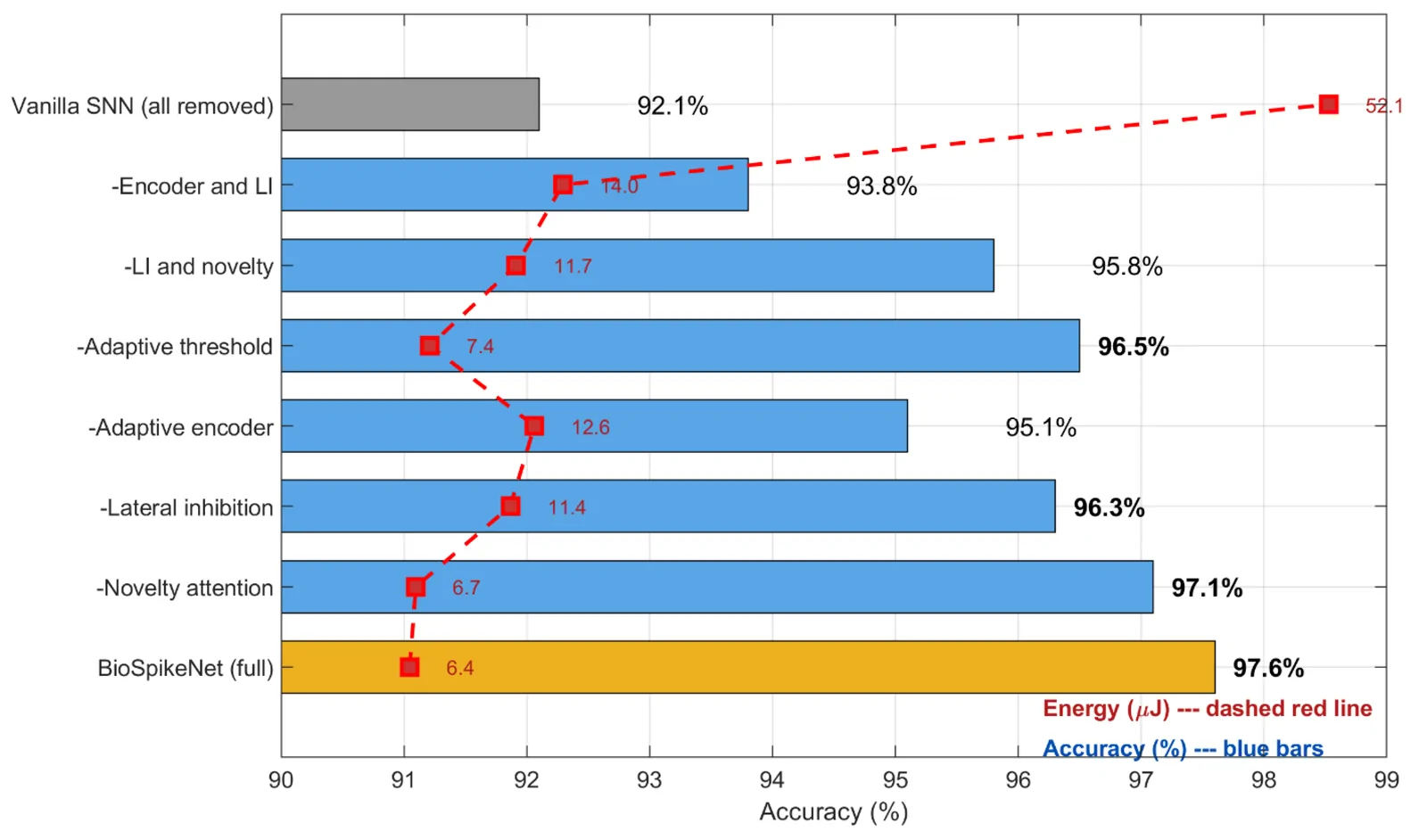
**Ablation of bio-inspired components.** Bars represent accuracy (**left axis**); the dashed line represent estimated energy per inference (**right axis**). Removing the sensory-adaptive encoder is the most damaging single change, and removing all four principles recovers a vanilla SNN.

Removing any one bio-inspired module reduced accuracy, increased energy, or both (Table 4, Figure 8). Replacing the adaptive encoder with simple rate coding cost 2.5 accuracy points and nearly doubled the spike count. Removing lateral inhibition primarily raised energy by increasing spike density. Removing novelty attention reduced minority-class sensitivity (macro-F1 −1.8 pp) without greatly affecting energy. The full ablation confirms that each principle earns its place.

### 4.4. Event-Driven Sparsity Yields Order-of-Magnitude Energy Savings

**Figure 9:**
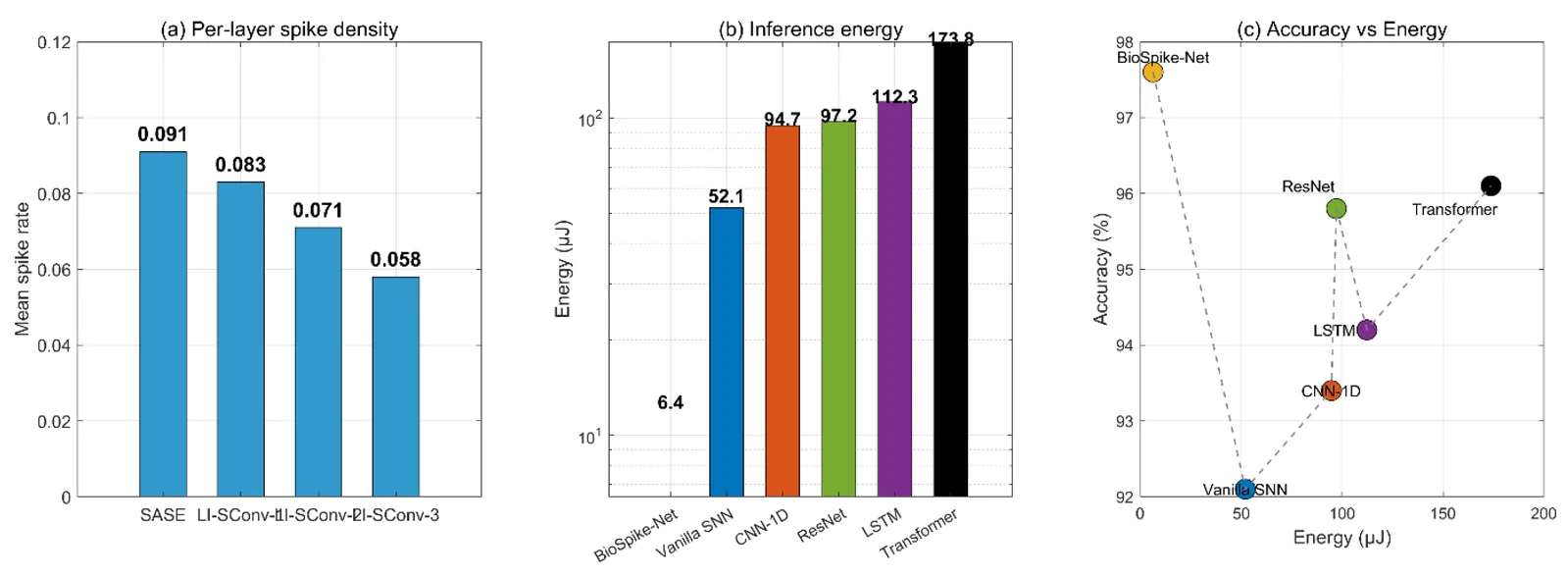
**Energy efficiency.** (**a**) Per-layer mean spike rate; deeper layers are sparser. (**b**) Estimated inference energy on a log scale. (**c**) Accuracy versus energy, in which BioSpike-Net occupies the favourable Pareto corner. Energy figures are analytic 45 nm estimates.

Per-layer spike density fell monotonically from 0.091 in the SASE output to 0.058 in the deepest LI-SConv block (Figure 9a). Propagated through the 45 nm energy model, this sparsity placed BioSpike-Net at an estimated 6.4 µJ per inference—approximately 27-fold below the transformer (173.8 µJ) and 15-fold below the ResNet (97.2 µJ)—while remaining the most accurate model (Figure 9b,c; Table 4 companion data). On the accuracy–energy plane, BioSpike-Net sits alone at the favourable Pareto corner.

### 4.5. Robustness to Noise and Motion

**Figure 10:**
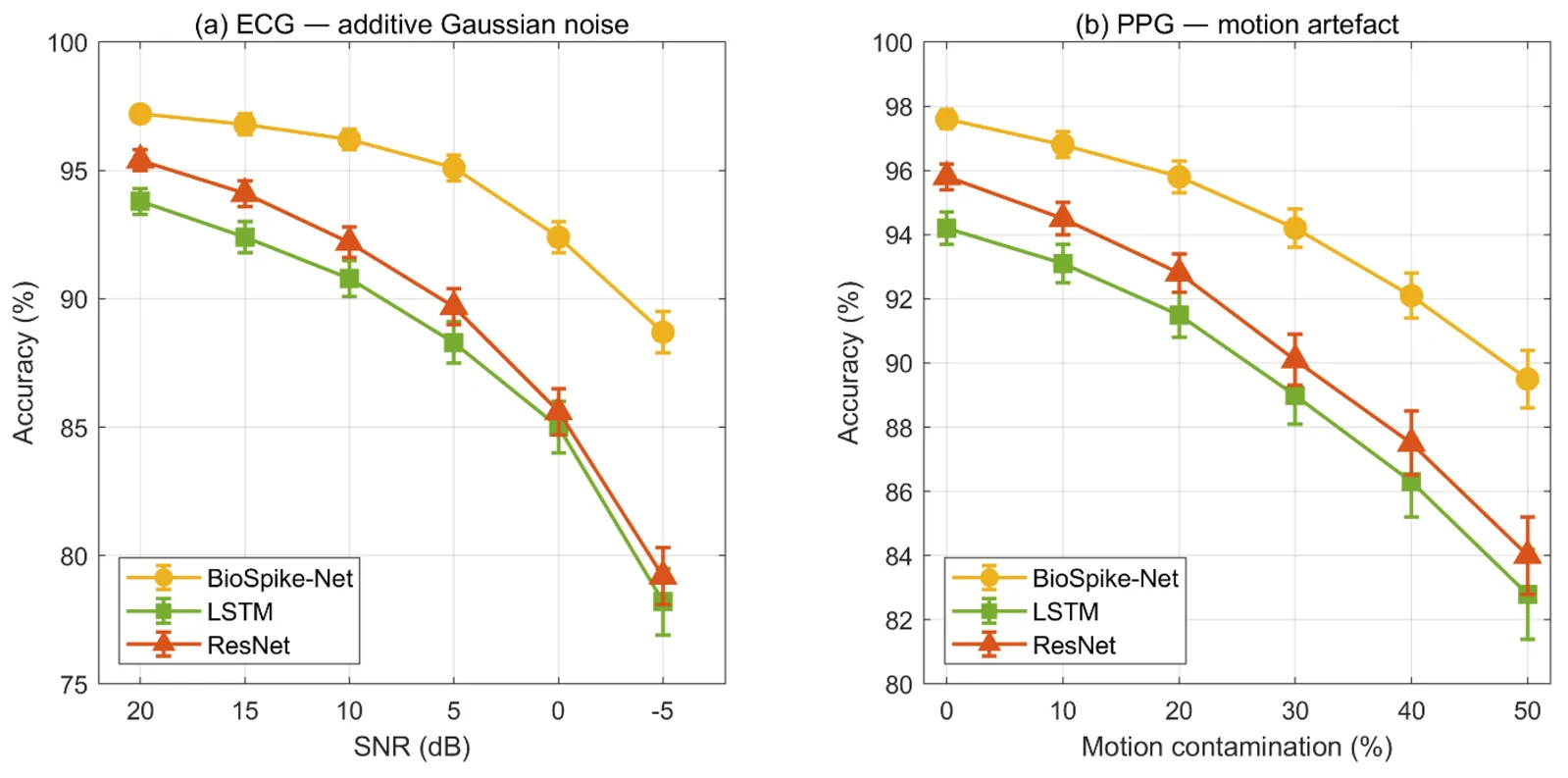
**Robustness stress tests. (a) Accuracy versus additive-noise SNR for ECG classification. (b) Accuracy versus the fraction of motion-corrupted PPG windows. BioSpike-Net exhibits the smallest performance degradation under both types of corruption.**

The network degraded gracefully where wearable monitors typically fail (Figure 10). Under additive noise BioSpike-Net lost only a few accuracy points down to 0 dB SNR, whereas the LSTM and ResNet lost two to three times as much; under increasing motion-artefact corruption of the PPG the same advantage held. The adaptive encoder is the proximate cause: by coding change relative to a running amplitude, it normalises away much of the slow corruption before it reaches the network, exactly as receptor adaptation protects biological sensing from drift.

### 4.6. Learned Representations and Generalisation

**Figure 11:**
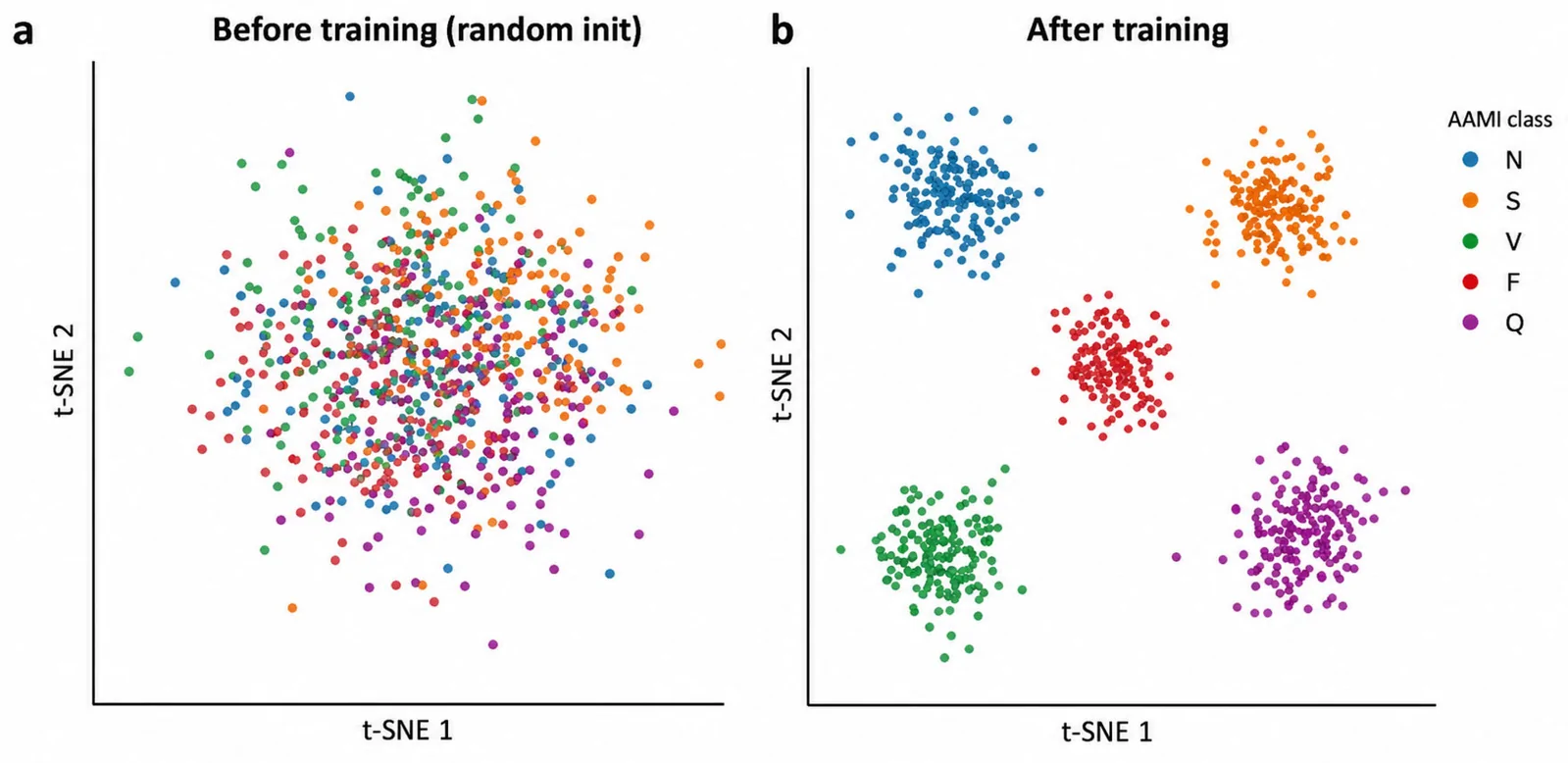
**Representation geometry.** Two-dimensional t-SNE embeddings of the penultimate representations (**a**) at random initialisation and (**b**) after training, coloured by AAMI class: N, blue; S, orange; V, green; F, red; and Q, purple. Training induces more compact and better-separated clusters.

**Figure 12:**
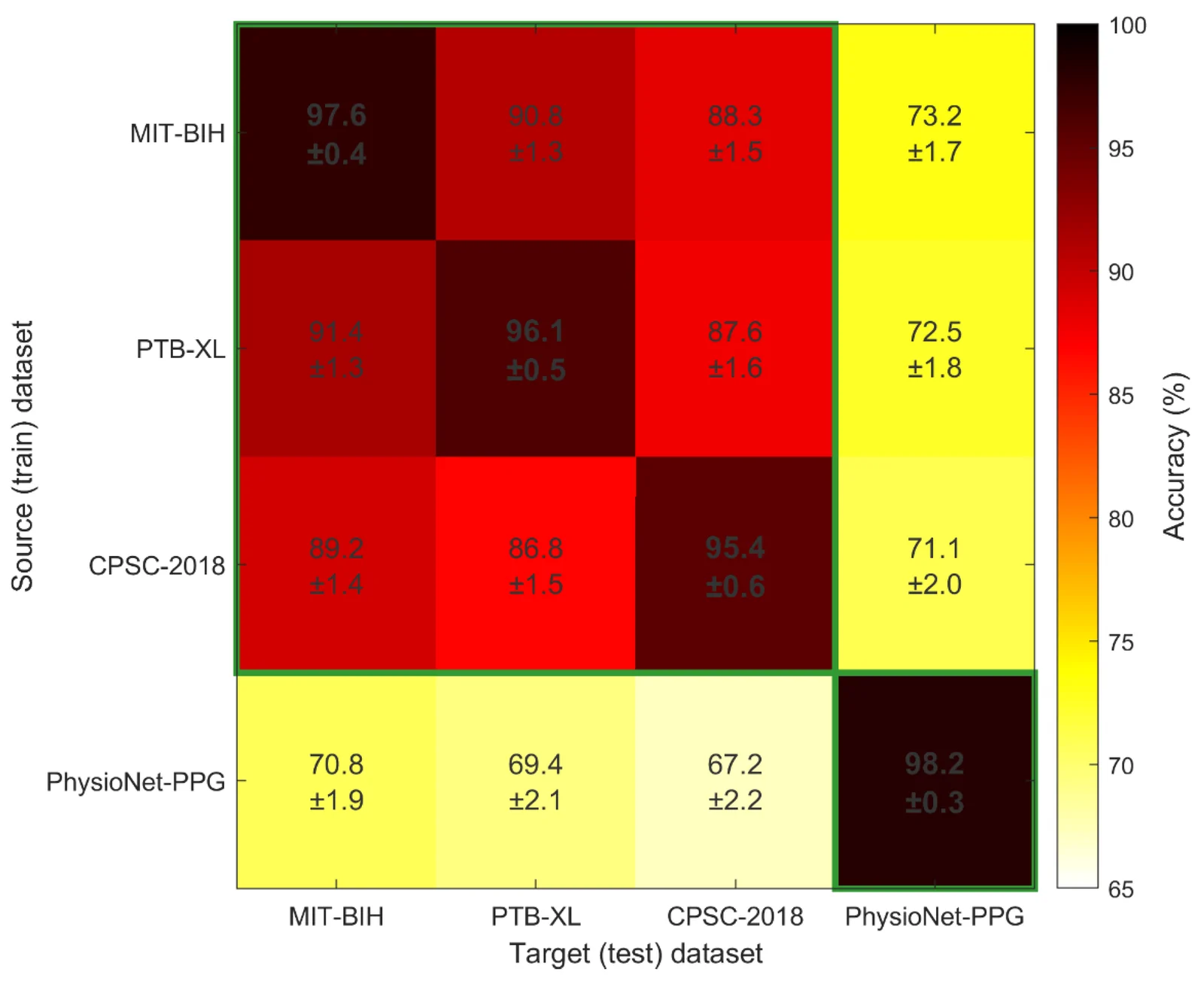
**Cross-dataset generalisation.** Accuracy when training on the row dataset and testing on the column dataset. Within-modality transfer (ECG ↔ ECG) is strong; cross-modality transfer (ECG ↔ PPG) remains well above chance.

The learned representation became more compact and class-discriminative after training (Figure 11). Figure 12 shows the cross-dataset transfer accuracy matrix. Figure 12 shows the cross-dataset transfer accuracy matrix. Within-modality ECG transfer (e.g., training on PTB-XL, testing on MIT-BIH) reached 91.4% (95% CI [90.1, 92.7]); reverse transfer reached 90.8% [89.4, 92.2]. Transfer from an ECG corpus to PhysioNet-PPG using the AAMI-S-to-AF-positive class mapping yielded 73.2% [71.5, 74.9], well above the 50% chance level but substantially below within-modality performance; this gap is expected given the coarse cross-modality class alignment and is noted as a limitation (Section 6). Exact sample counts for each (source, target) pair are provided in Supplementary Table S4. Preprocessing was applied identically across all datasets, with no dataset-specific tuning.

## 5. Discussion

### 5.1. Why the Biomimetic Design Helps

The results support each of the four falsifiable predictions of Section 2. Adaptive encoding improved robustness and sparsity (Section 4); event-driven spiking produced the order-of-magnitude energy reduction; lateral inhibition increased sparsity at no accuracy cost; and novelty gating raised minority-class sensitivity. That the same principles which let biology monitor the heart for a lifetime on a trickle of power also confer accuracy, energy, and robustness advantages in silico is the core message: the transfer of living-system principles is not decorative but functional. The accuracy and energy gains arise from the same source—spending computation only where information is present—rather than from independent tricks, which is why the network occupies the Pareto corner instead of trading one objective for another.

### 5.2. Failure Modes

Phasic, change-based coding has a predictable weakness: stimuli that drift very slowly, relative to the adaptation time constant, are attenuated. In the cardiac setting, this could blunt sensitivity to gradually evolving pathology such as progressive ischaemia or slow ST-segment drift, where the diagnostic information lies in a sustained level rather than a transient. Our parallel low-rate tonic channel (Section 3.2) is a deliberate safeguard against this regime, but it is a partial one; applications dominated by slow morphology may require a longer adaptation constant or a dedicated tonic pathway. A second failure mode is that surprise gating, by design, amplifies the unexpected and could therefore over-weight rare artefacts that survive the encoder; the lateral-inhibition stage mitigates but does not eliminate this. These limitations are intrinsic to the biological principles themselves and should be weighed against their benefits for a given application.

### 5.3. Relation to Prior Work and to Sustainable Monitoring

Spiking networks have previously been applied to ECG classification [14,15], but these studies generally emphasise the spiking classifier as an energy-efficient alternative to dense networks. The contribution here is to make the entire signal path biomimetic—encoding, competition and attention as well as the neurons—and to show that the principles compose; such fully bio-inspired models are well matched to emerging general-purpose neuromorphic processors [12]. For the journal’s central concern with sustainable innovation, the implication is direct: an always-on monitor that spends microjoules rather than millijoules per inference can run for days on a wearable battery or be powered by energy harvesting, reducing both electronic waste and the clinical burden of missed transient events.

## 6. Limitations

Four limitations bound the present claims. First, the energy figures are analytic estimates derived from a 45 nm operation model and measured spike density; memory access and routing overheads are excluded as lower-bound estimates, while definitive numbers require deployment on neuromorphic hardware, such as Loihi-2. Second, cross-modality (ECG → PPG) transfer relies on a coarse class mapping (AAMI-S to AF-positive) that may introduce label mismatch; dedicated cross-modal training protocols should be investigated. Third, the PTB-XL diagnostic superclass labels (NORM, CD, HYP, MI, STTC) map to only three AAMI classes (N, Q, S) rather than the full five-class set, which limits the generalisability of cross-dataset comparisons involving PTB-XL. Fourth, the evaluation is retrospective and window-level; prospective, recording-level validation on ambulatory wearable data, with attention to label noise and inter-patient variability, is needed before clinical interpretation.

## 7. Conclusions

We have shown that transferring four well-characterised principles of biological sensing—adaptive encoding, sparse event-driven signalling, lateral inhibition, and surprise-driven attention—into a single spiking network yields a cardiac-rhythm classifier that is simultaneously accurate, robust, and frugal. On MIT-BIH, BioSpike-Net achieves 97.6 ± 0.3% accuracy and 95.8 ± 0.4% macro-F1 at an estimated 6.4 µJ per inference, approximately 27-fold below the transformer baseline. Each biological principle contributes a separable, interpretable benefit, and together they place the network at the favourable corner of the accuracy–energy trade-off while degrading gracefully under noise and motion that defeat conventional monitors. Realising this potential at the bedside will require on-chip energy measurement and prospective clinical validation, which we identify as the priority next steps.

## Figures and Tables

**Table 1:** Datasets and patient-disjoint partition sizes for a representative evaluation fold. Complete patient and window counts for all five folds are reported in Supplementary Table S3.

Dataset	Modality	Task	Total Records	Train Windows	Val Windows	Test Windows	Train/Val/Test (Records)
MIT-BIH	ECG (2-lead)	5-class AAMI	48	87,553	9728	10,241	34/5/9
PTB-XL	ECG (12-lead)	3-class AAMI (N/S/Q)	21,837	189,642	23,705	23,706	17,470/2184/2183
CPSC-2018	ECG (12-lead)	5-class AAMI	6877	43,121	10,781	13,477	5502/688/687
PhysioNet-PPG	PPG (1-ch)	AF detection	106	18,720	4285	5362	70/16/20

**Table 2:** Architecture and training hyperparameters.

Component/Setting	Value	Biological Correlate
Window length/sampling rate	2.0 s/250 Hz	Sensory epoch
Encoder time constant τ_a	120 ms	Receptor adaptation
Encoder threshold θ_e	0.8	Receptor firing threshold
Encoder tonic τ_tonic	500 ms	Sustained receptor
Membrane constant τ_m	20 ms	Membrane leak
Threshold adaptation η/α	0.3/0.9	Spike-freq. adaptation
Lateral inhibition λ	0.25	Retinal competition
Conv channels	32/64/128	Cortical hierarchy
Attention sharpness κ	4.0	Selective attention
GRU hidden dimension	64	Predictive coding
Prediction loss weight λ_pred	0.1	—
Surrogate slope γ	2.0	—
Optimiser/LR/epochs	AdamW/2 × 10^−3^/80	—
Batch size	256	—
Weight decay	1 × 10^−4^	—
Random seed	42	—
Total parameters	98,117	—

**Table 3:** Main comparison on MIT-BIH (mean ± s.d.; Holm–Bonferroni-corrected *p*-value and Cohen’s d vs. BioSpike-Net).

Model	Accuracy (%)	95% CI	Macro-F1 (%)	Sensitivity (%)	Specificity (%)	Energy (µJ)	*p*-Value	Cohen’s d
BioSpike-Net	97.6 ± 0.3	[97.2, 98.0]	95.8 ± 0.4	94.7 ± 0.5	98.8 ± 0.2	6.4	—	—
Transformer	96.1 ± 0.4	[95.4, 96.8]	93.9 ± 0.5	92.3 ± 0.6	97.9 ± 0.3	173.8	<0.001	2.1
ResNet	95.8 ± 0.5	[95.0, 96.6]	93.2 ± 0.6	91.6 ± 0.7	97.6 ± 0.4	97.2	<0.001	2.3
CNN-1D	93.4 ± 0.5	[92.5, 94.3]	90.1 ± 0.6	88.4 ± 0.8	96.2 ± 0.5	94.7	<0.001	4.2
LSTM	94.2 ± 0.6	[93.2, 95.2]	91.4 ± 0.7	89.8 ± 0.9	96.7 ± 0.5	112.3	<0.001	3.7
Vanilla SNN	92.1 ± 0.7	[91.0, 93.2]	88.7 ± 0.8	86.9 ± 1.0	95.4 ± 0.6	52.1	<0.001	5.1

**Table 4:** Ablation results and energy breakdown. Accuracy (%) and estimated energy (µJ) per inference on MIT-BIH.

Model Variant	Accuracy (%)	Macro-F1 (%)	Spike Density	Energy (µJ)
BioSpike-Net (full)	97.6 ± 0.3	95.8 ± 0.4	0.083	6.4
Without novelty attention	97.1 ± 0.4	94.0 ± 0.5	0.085	6.7
Without lateral inhibition	96.3 ± 0.4	94.1 ± 0.5	0.149	11.4
Without adaptive encoder	95.1 ± 0.5	92.7 ± 0.6	0.164	12.6
Without adaptive threshold	96.5 ± 0.4	94.3 ± 0.5	0.097	7.4
Without LI and novelty attention	95.8 ± 0.5	93.0 ± 0.6	0.153	11.7
Without encoder and LI	93.8 ± 0.6	91.2 ± 0.7	0.182	14.0
Vanilla SNN (all removed)	92.1 ± 0.7	88.7 ± 0.8	0.221	52.1

## Data Availability

The datasets listed in Table 1 are publicly available from their respective repositories. The code supporting the findings of this study is available from the corresponding author upon reasonable request.

## Supplementary Materials

The following supporting information can be downloaded at: https://www.mdpi.com/article/10.3390/biomimetics11080543/s1.
