# Supplementary material for: Neuromorphic Cardiac Sensing: A Bio-Inspired Spiking Neural Network with Sensory-Adaptive Encoding for Energy-Efficient Arrhythmia Detection from ECG and PPG Signals
Source: Biomimetics (Basel). 2026 Aug 3;11(8):543. doi: 10.3390/biomimetics11080543 (PMC13509974; doi:10.3390/biomimetics11080543)
Supplement: Supplementary file 1 [file biomimetics-11-00543-s001.zip › Tables_S1_S4.pdf]

Supplementary Materials for BioSpike-Net

Table S1: Class distributions per dataset split (window counts)

Dataset	Split	N	S	V	F	Q
MIT-BIH	Train	62,437	10,248	8,931	3,104	2,833
MIT-BIH	Val	6,945	1,139	993	345	306
MIT-BIH	Test	7,312	1,199	1,045	363	322
CPSC-2018	Train	30,620	5,601	3,104	2,145	1,651
CPSC-2018	Val	7,658	1,400	777	536	410
CPSC-2018	Test	9,570	1,751	970	669	517

Dataset	Split	N	S	Q
PTB-XL	Train	146,784	22,512	20,346
PTB-XL	Val	18,348	2,815	2,542
PTB-XL	Test	18,350	2,813	2,543

Dataset	Split	Non-AF	AF
PhysioNet-PPG	Train	15,913	2,807
PhysioNet-PPG	Val	3,642	643
PhysioNet-PPG	Test	4,558	804

Table S2: Layer-wise energy breakdown for all models

Model	Layer	Op type	Spike density	Energy const.	Energy (nJ)
BioSpike-Net	SASE encoder	AC-equiv	0.091	E_AC = 0.9 pJ	0.02
BioSpike-Net	LI-SConv-1	AC	0.083	E_AC	0.84
BioSpike-Net	LI-SConv-2	AC	0.071	E_AC	2.18
BioSpike-Net	LI-SConv-3	AC	0.058	E_AC	2.91
BioSpike-Net	Readout	MAC	—	E_MAC = 4.6 pJ	0.45
BioSpike-Net	Total	—	—	—	6.40
Vanilla SNN	Conv-1 (dense)	MAC	0.221	E_MAC	20.8
Vanilla SNN	LIF-1 (T=20)	AC	0.221	E_AC	15.8
Vanilla SNN	Conv-2 (dense)	MAC	0.221	E_MAC	8.3
Vanilla SNN	LIF-2 (T=20)	AC	0.221	E_AC	5.6

Vanilla SNN	Readout	MAC	—	E_MAC	1.6
Vanilla SNN	Total	—	—	—	52.1
CNN-1D	3 Conv + FC	MAC	—	E_MAC	94.7
ResNet-1D	Stem + 3 Res + FC	MAC	—	E_MAC	97.2
LSTM	2x LSTM + FC	MAC	—	E_MAC	112.3
Transformer	Enc + Attn + FC	MAC	—	E_MAC	173.8

Table S3: Per-fold metrics on MIT-BIH (accuracy %, Macro-F1 %, patients, windows)

Fold	BioSpikeNet Acc	B-F1	Transf Acc	T-F1	ResNet Acc	R-F1	CNN Acc	C-F1	LSTM Acc	L-F1	Patients	Windows
1	97.4	95.6	96.0	93.7	95.6	93.0	93.2	89.9	94.0	91.2	7	2,048
2	97.8	96.0	96.3	94.1	96.0	93.4	93.6	90.3	94.4	91.6	7	2,048
3	97.3	95.5	95.8	93.5	95.5	92.8	93.1	89.7	93.8	91.0	7	2,048
4	97.9	96.2	96.5	94.3	96.2	93.6	93.8	90.5	94.6	91.8	7	2,048
5	97.6	95.7	95.9	93.9	95.7	93.2	93.3	90.1	94.2	91.4	7	2,049

Table S4: Cross-dataset transfer — sample counts and 95% bootstrap CIs

Source (Train)	Target (Test)	Source windows	Target windows	Accuracy (%)	95% CI
MIT-BIH	PTB-XL	107,522	23,706	90.8	[89.4, 92.2]
MIT-BIH	CPSC-2018	107,522	13,477	88.3	[86.5, 90.1]
MIT-BIH	PPG	107,522	5,362	73.2	[71.5, 74.9]
PTB-XL	MIT-BIH	237,053	10,241	91.4	[90.1, 92.7]
PTB-XL	CPSC-2018	237,053	13,477	87.6	[85.8, 89.4]
PTB-XL	PPG	237,053	5,362	72.5	[70.6, 74.4]
CPSC-2018	MIT-BIH	67,379	10,241	89.2	[87.4, 91.0]
CPSC-2018	PTB-XL	67,379	23,706	86.8	[84.9, 88.7]
CPSC-2018	PPG	67,379	5,362	71.1	[68.8, 73.4]
PPG	MIT-BIH	28,367	10,241	70.8	[68.5, 73.1]
PPG	PTB-XL	28,367	23,706	69.4	[67.0, 71.8]
PPG	CPSC-2018	28,367	13,477	67.2	[64.5, 69.9]
